# Henry Clay

**Published:** 1879-05

**Authors:** 


### HENRY CLAY.

NEAR three hundred years ago (1584) Sir Walter Raleigh brought a company of colonists over to Virginia, among whom were the three sons of Sir John Clay, of Wales, to each of whom the father had given fifty-thousand dollars; their names were Charles, Thomas, and Henry, the last had no children.

Cassius M. Clay is a descendant of Charles; Henry Clay and Porter, were descendants of Thomas. Thus, Charles and Thomas Clay, sons of Sir John, are the progenitors of all the Clays in the United States.

The father of Henry and Porter Clay, was a Baptist Clergyman, (and so was Porter Clay himself) and left no heirs of his name except Henry, and Porter Clay.

All of Henry Clay's daughters (six) died; of his five sons, the eldest, Theodore, was a lunatic. Henry Clay, Jr., killed at the battle of Bueno Vista, left three children.

